# The Value of Family History in Colorectal Screening Decisions for Oldest Old Geriatric Populations

**DOI:** 10.7759/cureus.12815

**Published:** 2021-01-20

**Authors:** David R Miller, Leon Averbukh, Gurjiwan Virk, Mikram Jafri, Micheal Tadros

**Affiliations:** 1 Gastroenterology, Albany Medical Center, Albany, USA; 2 Department of Medicine, Division of Gastroenterology, Allegheny Health Network, Pittsburgh, USA; 3 Internal Medicine, Albany Veterans Affairs Stratton Medical Center, Albany, USA

**Keywords:** colorectal cancer, screening colonoscopy, elderly, family history, cancer screening

## Abstract

Introduction: Colorectal cancer (CRC) is the second most common form of cancer affecting both men and women. Extensive screening guidelines have been developed to help reduce the incidence of disease. Currently, United States Preventative Service Task Force guidelines recommend against routine screening in those 85 years and older. However, octogenarians and nonagenarians continue to be screened for CRC with no consensus on indications. The aim of this study is to examine family history of CRC as a risk factor and clinical indication for providing screening colonoscopies to the “oldest old” geriatric population, defined as aged 80 years and above.

Methods: We conducted a retrospective review of a Veterans’ Health Administration database to identify male veterans aged 80 years and older who underwent screening colonoscopy. Subsequently, we examined those who tested positive for CRC with a family history of CRC.

Results: Of the 458,224 patients who are 80 years and older in the Veterans Affairs (VA) database, 17.8% underwent a screening colonoscopy; 11.42% of these individuals were further diagnosed with CRC; and 8.89% of those with diagnosed CRC had a documented family history of CRC.

Conclusion: Family history should not be used as an inclusionary criterion for CRC screening in the 80 years and above age group as the rate of CRC in these patients with a family history of CRC is significantly lower than that in the younger age groups with a family history of CRC.

## Introduction

Approximately 135,430 cases of colorectal cancer (CRC) were diagnosed in the United States in 2017, with a further 50,260 deaths from CRC-related illness [1]. Of the roughly 50,260 reported deaths, approximately 16,730 were classified in “oldest old” (those who are above 80 years old) category by the World Health Organization [1]. Despite being the second most common cause of cancer that affects both men and women, the mortality rate of the disease was noted to be steadily declining between the years 2000 and 2014 [2]. This decrease in CRC mortality rate may be explained by more effective screening practices and guidelines as well as advancing therapeutic options for treatment of CRC [3].

The United States Preventative Services Task Force (USPSTF) currently recommends CRC screening for patients between the ages of 50 and 75 years [4]. However, for those above the age of 75 years, the USPSTF recommends using a more nuanced approach to screening decision-making, which includes considering overall patient health and estimated remaining lifespan as well as the patient’s prior CRC screening history. Ultimately, only if the patient is estimated to have a significant lifespan remaining and has not been screened for CRC prior to age 76, they should undergo CRC screening [4]. Another organization, the United States Multi-Society Task Force on Colorectal Cancer, recommends CRC screening for patients without prior screening up to age of 85 years [5]. For those above the age of 85 years, major CRC screening guidelines recommend against screening those with deemed average risk for CRC irrespective of prior screening [6].

While the aforementioned guidelines have generally been well received by the medical community, there still remains a lack of a clear consensus concerning the utility of CRC screening in patients aged 76 years and older. Using a cost-effectiveness analysis, van Hees et al. showed that CRC screening for the elderly is beneficial up to ages 86, 83, and 80 years when testing for the variables “no comorbidity,” “moderate comorbidity,” and “severe comorbidity,” respectively [7]. Tang et al. concluded that patients with a life expectancy of ≥10 years may benefit from screening sigmoidoscopy [8]. Additionally, despite the USPSTF recommendation to not routinely screen for CRC in patients aged ≥76 years, a significant number of these patients are still being screened for CRC. Virk et al. reported that from the years 2000 to 2015, 81,946/458,224 (17.8%) of male veterans aged ≥80 years received colonoscopies in the United States Veterans Affairs (VA) healthcare system [9].

Interpreting the available literature in addition to following current official recommendations of task groups and professional organizations suggests that age should not be the sole deciding factor when considering whether to perform CRC screening in the elderly. Thus, it appears that clinicians are weighing additional factors such as patient comorbidities, overall patient health, and family history of CRC when deciding to screen patients above the age of 75 years.

When considering a patient's family history of CRC for deciding to screen the patient for CRC, it is important to recognize current estimates that indicate a two-fold increase of being diagnosed with CRC if a first-degree relative under 60 years has CRC [10]. This understanding has led to a firm consensus statement by the American Gastroenterological Association and the Canadian Association of Gastroenterology to strongly recommend CRC screening for all individuals with a family history of CRC [11]. The utility of a positive family history of CRC and the role it plays in deciding to screen for CRC are well established in younger populations; however, little data exists to support such practices in patients aged ≥80 years. We investigated this question using a dataset of male veterans at the VA aged ≥80 years who received colonoscopies from the years 2000 to 2015.

## Materials and methods

We performed a retrospective chart review on US male veterans aged 80 years and older from years 2000 to 2015 using a Veterans’ Health Administration database called the VA Informatics and Computing Infrastructure (VINCI) database.

A cohort of patients receiving diagnostic and screening colonoscopies was then identified using Current Procedural Technology (CPT) codes 45378-45398, G0105, and G0121 [12]. We excluded female veterans as there were very few who received colonoscopies during this time period. In our final cohort of patients, we applied International Statistical Classification of Diseases, Ninth Revision (ICD-9) codes 153.0-153.9 and 154.0 (which describe CRC in more specific locations) to identify those diagnosed with CRC. These patients were then placed into three age groups (Group 1: 80-84 years old, Group 2: 85-90 years old, and Group 3: >90 years old) and tallied using functions in Microsoft Excel. We then calculated the proportion of patients diagnosed with CRC with and without a documented family history of CRC with subsequent data analysis using chi-squared test using XLSTAT 2017 software (Addinsoft, Paris, France).

To avoid duplicates, each patient had an individual identifier. Additionally, a single patient receiving multiple colonoscopies was only counted as one colonoscopy by the VINCI algorithm.

**Figure 1:**
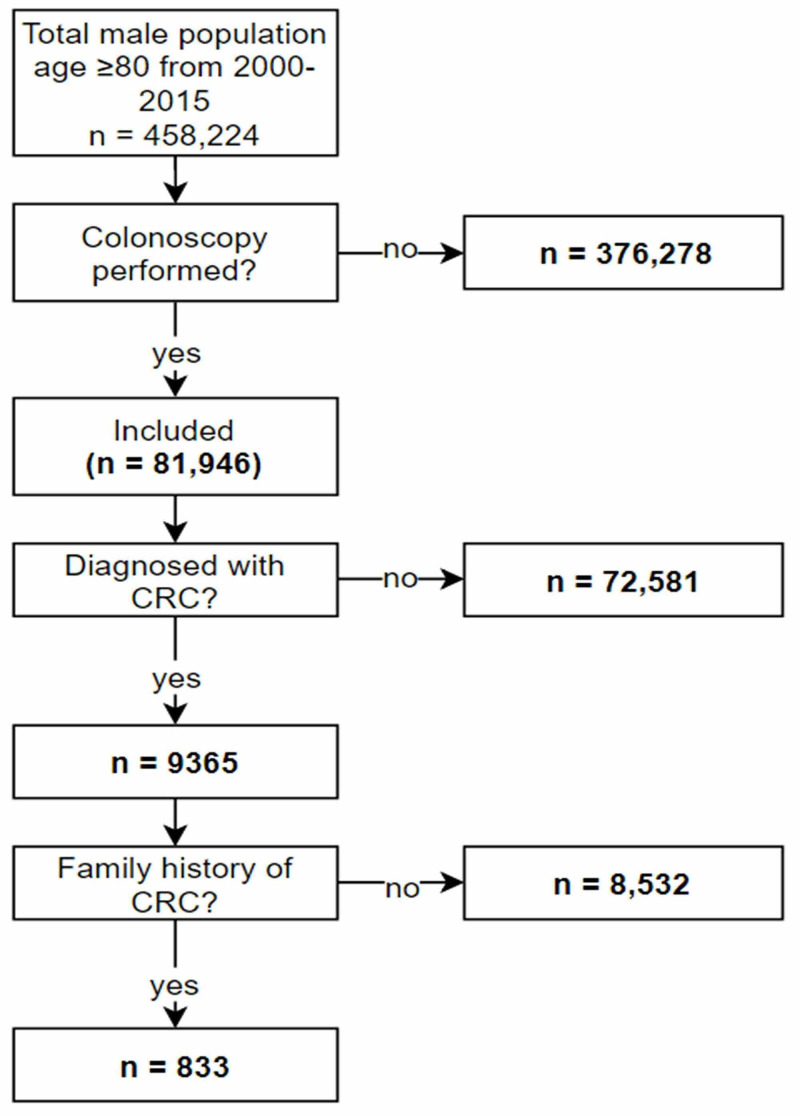
Flow diagram outlining selection of patient study population CRC, Colorectal cancer.

A flowchart describing our target patient identification and cohort construction is shown in Figure 1.

This study was approved by the Samuel S. Stratton Veterans Affairs (VA) Medical Center Institutional Review Board (FWA #00002073; IRB #00000950). Informed consent was deemed not necessary to obtain as this study was conducted as a retrospective database review.

## Results

A total of 458,224 male patients were identified as being 80 years or older during the studied time interval (2000-2015); 81,946 of the 458,224 patients (17.8%) underwent a colonoscopy; and 9,365 of 81,946 (11.42%) were diagnosed with CRC (Figure 1). Eight hundred and thirty three of the 9365 patients diagnosed with CRC (8.89%) had a documented family history of CRC. Table 1 shows the total number of patients by age group (80-84 years, 85-90 years, and above 90 years) diagnosed with CRC both with and without a documented family history of CRC.

**Table 1:** Number and proportion of patients by age group diagnosed with CRC with and without a family history of CRC Values are reported as numbers (proportion in %). CRC, Colorectal cancer.

Age	CRC With a Family History of CRC	CRC With No Family History of CRC	Total
80-84 years	139 (7.35%)	1750 (92.65%)	1889
85-90 years	443 (9.92%)	4020 (90.08%)	4463
>90 years	251 (8.33%)	2762 (91.67%)	3013
Total	833 (8.89%)	8512 (91.1%)	9365

A chi-squared test between the three age groups, Groups 1 to 3, who had CRC and a family history of CRC revealed a statistically significant difference between the different age groups (p = 0.01).

## Discussion

Colonoscopy in patients aged ≥80 years is associated with increased risk of morbidity and complications; thus, careful consideration of the risks and benefits must be weighed by the physician and patient before performing a colonoscopy [13].

Despite recommendations to cease CRC screening after the age of 75 years, elderly patients should not forego colon cancer screening solely because of their age as 11.42% of patients who received screening colonoscopies were diagnosed with CRC. In our study population, patients receiving a colonoscopy who were diagnosed with CRC, 8.89% had a documented family history of gastrointestinal (GI) malignancy or colonic polyps. This number is consistent with a retrospective cohort study by Tran et al. who found a documented family history of CRC in 10.6% (514/4834) of patients aged ≥75 years undergoing surveillance colonoscopy [14].

In the general population, approximately 20%-30% of patients with CRC have a first-degree relative with colon cancer, according to familial clustering studies and twin concordance studies [15,16]. Given that this study population’s family history of CRC is lower than that of the general population, it suggests that presence of a family history of CRC is less of a valuable predictor of an increased risk of CRC in this group. It is possible that patients younger than 80 years with a family history of CRC had the benefit of pre-cancerous polyp removal from a prior screening colonoscopy and thus may have been protected from developing CRC. Alternatively, given that the average age of CRC diagnosis in males is 68 years, the presently studied cohort may have had fewer family members with CRC given that those diagnosed with CRC likely did not reach the advanced age presently studied [17].

When deciding whether to screen elderly patients for CRC, lack of a family history of CRC in “oldest old” age group should not discourage screening for CRC. The decision to undergo screening colonoscopy should be made using a risk-benefit analysis of the overall patient’s condition and their likelihood to benefit from screening. This type of assessment should also be applied to diagnostic colonoscopies for elderly patients with lower GI symptoms such as change in bowel habits, bowel caliber, bright red blood per rectum, and weight loss. 

One of the limitations of this study was that ICD codes for family history of CRC did not differentiate between the degree of relative with a history of CRC. This is important as the risk of individual CRC is based on the number and degree of relatives afflicted by the condition. Additionally, it is possible that some patients in the examined cohort did have a family history of CRC, which was not documented in the chart, resulting in an overall underreporting. Furthermore, veterans aged ≥65 years with Medicare may have received CRC screening at hospitals outside of the VA, which was never documented in their VA electronic health record. An additional limitation of this study was the exclusive use of males and the fact that possible confounding variables such as smoking status and ethnicity, which are factors known to affect risk of CRC, were not collected. Lastly, a family history of CRC was not investigated in patients who did not receive CRC screening and is worth investigating in a future study.

One interesting finding of this study was the result of the chi-square test between the three studied age groups, which showed a significant difference in the family history of CRC rates in those diagnosed with CRC. At this time, the significance of this finding is unclear.

## Conclusions

Ultimately, in this study population, the decision to undergo screening colonoscopy should be made using a risk-benefit analysis of overall patient condition and their likelihood to benefit from screening colonoscopy. The presence of a family history of CRC in patients aged ≥80 years should not be considered as a supporting factor to perform CRC screening. Further randomized controlled clinical trials are needed to elucidate the benefit and drawbacks of CRC screening in the elderly population.

## References

[1] Siegel RL, Miller KD, Fedewa SA, Ahnen DJ, Meester RGS, Barzi A, Jemal A (2017. CA Cancer J Clin. 2017). Colorectal cancer statistics.

[2] Jemal A, Ward EM, Johnson CJ (2017). Annual report to the nation on the status of cancer, 1975-2014, featuring survival. J Natl Cancer Inst.

[3] Baxter NN, Goldwasser MA, Paszat LF, Saskin R, Urbach DR, Rabeneck L (2009). Association of colonoscopy and death from colorectal cancer. Ann Intern Med.

[4] Bibbins-Domingo K, Grossman DC, Curry SJ (2016). Screening for colorectal cancer: US preventive services task force recommendation statement. JAMA.

[5] Rex DK, Boland CR, Dominitz JA (2017). Colorectal cancer screening: recommendations for physicians and patients from the U.S. multi-society task force on colorectal cancer. Am J Gastroenterol.

[6] Wolf AMD, Fontham ETH, Church TR (2018). Colorectal cancer screening for average-risk adults: 2018 guideline update from the American cancer society. CA Cancer J Clin.

[7] van Hees F, Habbema JDF, Meester RG, Lansdorp-Vogelaar I, van Ballegooijen M, Zauber AG (2014). Should colorectal cancer screening be considered in elderly persons without previous screening? A cost-effectiveness analysis. Ann Intern Med.

[8] Tang V, Boscardin WJ, Stijacic-Cenzer I, Lee SJ (2015). Time to benefit for colorectal cancer screening: survival meta-analysis of flexible sigmoidoscopy trials. BMJ.

[9] Virk GS, Jafri M, Ashley C (2019). Colonoscopy and colorectal cancer rates among octogenarians and nonagenarians: nationwide study of US veterans. Clin Interv Aging.

[10] Quintero E, Carrillo M, Gimeno-Garcia AZ (2014). Equivalency of fecal immunochemical tests and colonoscopy in familial colorectal cancer screening. Gastroenterology.

[11] Leddin D, Lieberman DA, Tse F (2018). Clinical practice guideline on screening for colorectal cancer in individuals with a family history of nonhereditary colorectal cancer or adenoma: the Canadian association of gastroenterology banff consensus. Gastroenterology.

[12] (2019). Colonoscopy - CPT Codes 45378-45398, G0105, G0121. https://www.asge.org/docs/default-source/coding/colonoscopy_2018-coding-sheet.pdf.

[13] Lin OS (2014). Performing colonoscopy in elderly and very elderly patients: risks, costs and benefits. World J Gastrointest Endosc.

[14] Tran AH, Ngor EWM, Wu BU (2014). Surveillance colonoscopy in elderly patients: a retrospective cohort study. JAMA Intern Med.

[15] Grady WM (2003). Genetic testing for high-risk colon cancer patients. Gastroenterology.

[16] Lichtenstein P, Holm NV, Verkasalo PK (2000). Environmental and heritable factors in the causation of cancer--analyses of cohorts of twins from Sweden, Denmark, and Finland. N Engl J Med.

[17] (2019). Colorectal Cancer Facts & Figures 2017-2019. https://www.cancer.org/content/dam/cancer-org/research/cancer-facts-and-statistics/colorectal-cancer-facts-and-figures/colorectal-cancer-facts-and-figures-2017-2019.pdf.

